# Editorial: The role of genetics studies in the discovery of new viruses and in the analysis of pathogeny of viral infections

**DOI:** 10.3389/fgene.2023.1240812

**Published:** 2023-07-04

**Authors:** Germán Ernesto Metz, María Soledad Serena, Pablo Enrique Piñeyro, Sonia Cheetham, Guillermo Giovambattista

**Affiliations:** ^1^ Laboratorio de Virología, Facultad de Ciencias Veterinarias, Universidad Nacional de La Plata, La Plata, Argentina; ^2^ CONICET-CCT La Plata, La Plata, Argentina; ^3^ Veterinary Diagnostic Laboratory, Veterinary Diagnostic and Production Animal Medicine, Iowa State University, Ames, IA, United States; ^4^ School of Veterinary Medicine, St. George’s University, St. George’s, Grenada; ^5^ CONICET Institute of Veterinary Genetics (IGEVET), La Plata, Argentina

**Keywords:** viral pathogenesis, new viruses, genetics studies, SARS-CoV-2, genome wide analysis, veterinary viruses, human viruses, bioinformatic analysis

Following the development of the PCR and the internet in the early 80’s, the molecular revolution took off in the 90’s and by the new millennium most research was utilizing some variation of the technique and platform. Subsequently, the amount of information grew exponentially allowing the scientific community to speed up discovery and it has continued ever since. However, in the last decade the application of new technologies and bioinformatic approaches has again enhanced the creation of data and allowed sharing to a magnitude never seen before.

At present the focus is on approaches that have moved research from the genomics to the transcriptomics, proteomics, metabolomics, etc., allowing for a better understanding of pathophysiologic processes but also permitting the steep acceleration in drug discovery by permitting *in silico* modeling and prediction before moving into the lab, cutting costs and times significantly. Due to the exponential gain of knowledge on viral pathogenesis, we are on the verge of a new therapeutics’ revolution.

In recent years, we have seen the emergence of new viral infections such as SARS-CoV-2 and the re-emergence of known diseases such as Dengue or the continuous occurrence of spillover events of Influenza in different parts of the world. Globalization of human travel and encroachment into natural environments have increased opportunities for spillover viruses to travel around the globe.

Some of the works listed in this editorial depict ways in which some of the newer approaches are being applied to a broad variety of topics, from viral association in human cancer (Shen et al.) potential SARS-COV-2 antiviral targets (Park and Moon), elucidate the viral pathogenesis (Al-Kubati et al.; Jiang et al.; Liu et al.; Wei et al.) or applied to viral diseases of veterinary importance (O'Donoghue et al.).

From the initial attachment to the final release of viruses from infected cells, there is an active cross-talk between viral and host factors that determine the pathogenicity of viral infection. Wei et al. demonstrated the pathogenicity of a fowl adenovirus isolated from the liver of chickens with hydropericardium hepatitis syndrome in China and also characterized the isolation by molecular and phylogenetic studies. On the other hand, O'Donoghue et al. investigated via RNA-sequencing (RNA-Seq) the transcriptome analysis of dairy calves experimentally challenged with Bovine Herpes Virus type 1 (BoHV-1) and compared the gene expression results with Bovine Respiratory Syncytial Virus (BRSV). Their genetics analysis identified Differentially Expressed Genes (DEGs) in infected and control animals that could identify potential therapeutic targets for bovine respiratory infection and also could assist in a selective breeding program in order to reduce disease susceptibility. The magnitude of data being generated by these approaches, as well as the data that can easily be additionally retrieved through the use of tools such as Gene Ontology is so vast that most studies have to select the top 3 or 5 DEGs and deposit the rest of the information on some supplementary file to maybe be investigated at a later time. The use of pathway enrichment analysis has permitted further understanding of the processes involved and the prediction of key network regulators, potential biomarkers and drug targets.

In the work by Al-Kubati et al. the researchers used immunoinformatics, machine learning, and artificial intelligence, to predict the virulence and immunogenicity of Bovine Viral Diarrhea Virus (BVDV) proteins, and they proposed to use this approach for developing novel diagnostic assays and vaccines. Similarly, Shen et al. used bioinformatics analysis to identify potential molecular biomarkers involved in EBV positive peripheral T cell lymphoma including transcription factors (TF), microRNAs (miRNAs) and Long Noncoding RNA (lncRNA). These studies present the recent interest in regulating molecules such as TF-mRNA, lncRNA-miRNA-mRNA, EBV-encoded miRNA-mRNA; how these can be from either from host or pathogen origin and how some of these regulatory functions may be used to inhibit viral replication or control cancer cells.

In relation to the new pandemic caused by SARS-CoV-2, Liu et al. employed a Mendelian randomization method. This method is widely used to examine the potential causal relationship between exposure and disease outcome. In this case, they explored the causality of myocardial injury in COVID-19 patients. They used single nucleotide polymorphisms (SNPs) as instrumental variables of exposure, finding that SARS-CoV-2 infection may exacerbate cardiac injury in these patients. Similarly, Jiang et al. described the use of a two-sample bidirectional Mendelian method and a genome-wide genetic correlation analysis based on the summary-level data of two large genome-wide association studies (GWAS). Their findings did not support the causal role of genetically predicted shorter leukocyte telomere length in the increased risk of COVID-19 phenotypes. Consequently, their study found no support for leukocyte telomere length as a biomarker for predicting COVID-19 outcomes.

Finally, Park and Moon identified 3′ of UTR mutations in the Alpha, Beta, Gamma, Delta SARS-CoV-2 variants that rarely occur as persistent mutations. It was revealed that the binding sites of the five miRNAs with antiviral effect were mostly conserved, even in newly occurring SARS-CoV-2 variants, so these miRNAs could be appropriate antiviral treatment despite the emergence of new variants.

Consequently, this Research Topic highlights the importance of genetic studies as versatile tools for analyzing viral pathogenesis and predicting the zoonotic potential of new viral strains. Moreover, such studies can determine the viral cellular pathogenesis and identify new treatments or preventive strategies against viral infection routes.

The threat of new viral infections continues and thus, *in vitro, in vivo* or *in silico* genetic studies must focus on trying to be prepared for the next pandemic viral strain. The increasing computer power together with the more recent implementation of artificial intelligence (AI) is revolutionizing this area of research. It is imperative that we prepare for the resulting products of such efforts and that we keep an overview on the whole process.

